# Emotional Intelligence and Its Relationship with Emotional Well-Being and Academic Performance: The Vision of High School Students

**DOI:** 10.3390/children7120310

**Published:** 2020-12-20

**Authors:** María Dolores Toscano-Hermoso, Carlos Ruiz-Frutos, Javier Fagundo-Rivera, Juan Gómez-Salgado, Juan Jesús García-Iglesias, Macarena Romero-Martín

**Affiliations:** 1 Department of Nursing, Faculty of Nursing, University of Huelva, 21007 Huelva, Spain; mdolores.toscano@denf.uhu.es (M.D.T.-H.); macarena.romero@denf.uhu.es (M.R.-M.); 2 Department of Sociology, Social Work and Public Health, Faculty of Labour Sciences, University of Huelva, 21007 Huelva, Spain; frutos@uhu.es (C.R.-F.); juanjesus.garcia@dstso.uhu.es (J.J.G.-I.); 3 Safety and Health Postgraduate Programme, Universidad Espíritu Santo, Guayaquil 091650, Ecuador; 4 Andalusian Health Service, Health Sciences Doctorate School, University of Huelva, 21007 Huelva, Spain

**Keywords:** emotional intelligence, personal well-being, adolescence, academic performance, high school, students

## Abstract

Emotional intelligence skills in students may be related with physical and mental health, within and outside the academic field. Strengthening these skills can lead to greater overall well-being, lower use of substances, and improved academic performance, as well as reduced aggressive behaviours. The objective of this study was to analyse the levels of emotional intelligence (differentiating between the dimensions: emotional Attention, Clarity, and Repair) among high school students and examine its relationship with academic performance and emotional well-being, considering if there are differences between boys and girls and between different grades. A cross-sectional descriptive study was developed on a sample of 333 High School students using the Trait Meta-Mood Scale (TMMS) and KIDSCREEN-10 Index tools. Differences in emotional intelligence were observed between boys and girls for the three dimensions, and a relationship between emotional intelligence and student well-being was appreciated. No relationships were found between emotional intelligence and academic performance, nor have any differences been observed between the different courses analysed. It cannot be concluded that academic performance is related to emotional intelligence, but a relationship between well-being and emotional intelligence is found.

## 1. Introduction

Adolescence is a stage in which a series of developmental changes occur that may threaten development and emotional well-being. This stage is especially characterised by certain instability, where the search for and consolidation of identity and the achievement of autonomy on the part of the adolescent is an important and necessary element of their lives [1]. On an emotional level, adolescents tend to be reserved regarding their problems and to not express their emotions or, on the other hand, to manifest them disproportionately. Thus, it becomes essential to encourage the development of emotional intelligence and encourage adolescents to observe and examine the feelings and emotions they experience [2].

The growing interest in the study of emotions over the past 30 years has led to numerous studies on emotional competences and their impact in various contexts [3,4]. In the early 1990s, the importance of emotional, personal and social aspects that could predict adaptation and success skills in life began to be emphasised, claiming a more global intelligence perspective, that is, emotional intelligence, defined as the ability to perceive, value, and express emotions accurately to access and/or generate feelings that facilitate thought, to understand emotions and emotional knowledge, and to regulate emotions by promoting emotional and intellectual growth.

Mayer and Salovey [5] designed one of the first instruments for measuring emotional intelligence, based on the three fundamental processes: perception, understanding, and regulation of emotions using the Trait Meta-Mood Scale (TMMS) [6,7].

From the concept of emotional intelligence, it can be extracted that it is closely linked to emotional well-being [5,8,9]. It has also been shown that both emotional and social development are important in academic performance to such an extent of finding statistically significant correlations between emotional intelligence and academic performance [10] in primary education [11]; secondary education [12,13,14,15,16]; as well as among university students [17,18]. These findings are justified by stating that emotional intelligence favours the deployment of essential socio-emotional skills for school learning and, on the other hand, reduce the possibility of disruptive behaviours that hinder academic learning [19]. However, there are other research works that contradict these results, finding null or very limited relationships between both variables [20,21]. The literature shows that gaps in emotional intelligence skills affect students inside and outside the school context. Specifically, emotional intelligence is related to physical and mental health [22,23,24,25], with greater well-being, lower substance use [26], better academic performance [12], as well as less aggressive behaviour [22].

In this sense, there are relationships between the various benefits of emotional education in the educational context such as: positive school coexistence; promotion of personal well-being; decreased rates of violence and aggression, of depressive symptomatology, anxiety and stress; improved school adaptation, self-esteem, and satisfactory personal relationships [27].

In Spain, according to the 2017 National Health Survey, 12.64% of the young population is at risk of poor mental health, 3.99% of suffering mental illness, 1.22% have depression, 2.06% have chronic anxiety, and 5.98% have had an appointment with the psychologist or psychotherapist in the last 12 months [28]. Navarro-Mateu et al.’s study revealed that the young Spanish population has a higher risk of suffering a mental health disorder, especially those related to anxiety [29]. Lapresa et al. pointed to a social component in the mental health of Spanish children, identifying that those born into less-favoured families have worse mental health [30]. According to Alonso-Fernández et al., the biggest difficulty that children in Spain find in relation to mental health is hyperactivity or attention deficit [31].

Therefore, the objectives of this study were, to analyse the levels of emotional intelligence (differentiating between the dimensions: emotional attention, clarity, and repair) among compulsory secondary education students (Spain) to find out if there are differences between boys and girls and between the grades and to examine their relationship with academic performance and emotional well-being.

## 2. Materials and Methods

### 2.1. Participants

A cross-sectional descriptive study was designed. The sample consisted of 333 students enrolled in the first, second, third, and fourth grade of Compulsory Secondary Education (ESO, for its acronym in Spanish) of the Colón Marist School, Huelva (Spain). Of the whole sample, 142 were girls (42.6%), and 191 boys (57.4%). The mean age of the participants was 14.03 years with a SD of 1.28. To obtain the study sample, non-probabilistic purposive sampling was used.

The Colón Marist School of Huelva is a private religious associated centre located near the downtown area of Huelva, with most students belonging to the downtown and periphery area, and compatible with medium-high and high socioeconomic levels. The family context coincides in an experience of faith as a mechanism to provide moral values which are considered important for the family and that are present throughout the educational process of students.

### 2.2. Instruments

The assessment of emotional intelligence was evaluated through the Trait Meta-Mood Scale (TMMS) in its reduced version adapted to Spanish by Fernández-Berrocal et al. [7]. It is a self-administered tool that measures the level of perceived emotional intelligence (PEI) from the individual’s own meta-knowledge of the emotional states. It consists of 24 items based on the three dimensions stated by Mayer and Salovey [5] theoretical model of emotional intelligence. These are: (a) emotional attention is about to what extent individuals tend to observe and think about their feelings and moods (e.g., *I pay a lot of attention to how I feel* and *I don’t think it’s worth paying attention to your emotions or moods*); (b) emotional clarity refers to the understanding of one’s emotional states (e.g., *I am usually very clear about my feelings and I can’t make sense out of my feelings)*; and (c) emotional repair refers to the individuals’ beliefs about their ability to regulate their feelings (e.g., *Although I am sometimes sad, I have a mostly optimistic outlook* and *When I become upset, I remind myself of all the pleasures in life*). Each dimension is evaluated by 8 items. The scale of responses ranges from: 1 = do not agree; 2 = moderately agree; 3 = quite agree; 4 = highly agree; and 5 = totally agree. The scores of the three subscales are classified into three ranges (To improve, Adequate and Excellent) and vary according to the gender of the participants [7]. As for the psychometric properties of the scale, the authors report a high internal consistency for each dimension: Attention α = 0.90; Clarity α = 0.90; and Repair α = 0.86 [7]. The internal consistency in this study, obtained through Cronbach’s alpha regarding the different subscales, has been: Attention α = 0.81; Clarity α = 0.83; Repair α = 0.79.

It is necessary to note that an overall PEI score is not obtained, as this instrument has not been designed for this purpose, so it is important to take this into account for the interpretation of the results. On the other hand, the instrument does not measure the entire mastery of the PEI trait, but only part of it, so some central dimensions of the PEI trait are left out of the information provided by this scale. The authors of the scale provide values (Table 1) to calculate the subject’s final scores in each of the three dimensions with different cut-off points for males and females, as there are differences in scores for each of these groups [32]. These levels of interpretation have been used in previous studies such as the one by Guerra-Bustamante et al. [33].

Emotional well-being was assessed through the KIDSCREEN-10 Index [34], a self-administered tool consisting of 10 items derived from the KIDSCREEN-27 questionnaire version. For its development, Rasch’s analysis of the extended version was applied, the results of which provided a one-dimensional index of health-related quality of life, which has been widely used as a measure of emotional well-being. Items are answered on a 5-point Likert-type scale based on the degree of agreement with the statement presented (e.g., Have you felt fit and well? and Have you got on well at school? and Have you been able to pay attention?). Total scores range from 10 to 50, and higher scores indicate higher levels of emotional well-being. The instrument showed good internal consistency (Cronbach’s alpha of 0.82) and good test-retest stability (r = 0.73; ICC = 0.72) [35]. The internal consistency obtained in this study resulted in a Cronbach’s alpha value of 0.783 (α = 0.783).

For the analysis of the academic performance variable, the grades obtained in the previous term (2nd term) were requested for the four subjects common to the four courses included, namely the following: (a) mathematics; (b) language; (c) social sciences/geography and history; and (d) biology/physics and chemistry. The response options were 1—fail; 2—sufficient; 3—good; 4—very good; 5—excellent. Finally, the academic performance variable was obtained by calculating the mean of these four subjects.

### 2.3. Procedure

First, a meeting was held with the centre’s management body to explain the objectives of the study, show the questionnaires, and ensure the confidentiality of the study. Once cooperation standards were agreed, a meeting took place with the coordinators of the cycles that included the selected courses in order to explain the objective of the study, as well as to specify the dates and times of data collection. During this meeting, the questionnaire to be completed by the students was shown to them and they were urged to express any doubts.

Data collection took place at the beginning of the third term of the academic year 2018/2019 after informing the participants on their participation in a study on adolescents’ well-being and ensuring confidentiality and anonymity.

### 2.4. Data Analysis

Descriptive statistics were presented as percentages and frequencies. The Kolmogorov–Smirnov test was used to determine whether data followed a normal distribution.

Descriptive statistics were used for the whole sample, boys and girls and students of each course, to describe the sample regarding emotional intelligence levels related with the emotional attention, clarity, and repair dimensions.

In addition, for the comparison of emotional intelligence levels according to the sex of the participants, the chi squared statistic test and its corresponding effect size test (Cramer’s V) were used. For the comparison between courses, chi squared was used as a significance test and the Tau-c coefficient as an effect size test. Comparison of column proportion was performed when the chi-squared test was not significant.

To analyse the relationship of each dimension of emotional intelligence with academic performance, tests of comparison of means were used, specifically one-factor ANOVAs. Cohen’s d was used as an effect size test for each crossing of two response values, according to the following criteria: from 0 to 0.19, negligible; 0.20 to 0.49, low effect size; 0.50 to 0.79, medium effect size; and, from 0.80, large effect size.

The study of the relationship between emotional intelligence and well-being was also carried out through one-factor ANOVAs and Cohen’s d effect size test; a post-hoc analysis was presented for those subcategories that had significant differences.

The statistical analysis was performed with the SPSS 25 programme, assuming a significance level of *p* < 0.05.

### 2.5. Ethical Aspects

Subjects’ participation has been completely voluntary, guaranteeing anonymity at all times. Additionally, the participants signed an informed consent for inclusion before they participated in the study. The questionnaires explained the study subject matter in detail and included the participant’s consent. Participants’ responses were recorded anonymously, and the information was treated confidentially, following the regulations in force (Organic Law 3/2018, of 5 December, on the Protection of Personal Data and guarantee of digital rights).

The study was conducted under the “Ethical Principles for Medical Research Involving Humans” contained in the latest version of the Helsinki Declaration (Fortress Amendment, Brazil, October 2013). This project has been approved by the Ethics Committee of the Colon Marist School in Huelva on 25 May 2019.

## 3. Results

### 3.1. Analysis of Emotional Intelligence Levels

It is observed that, of the total sample of 333 students, approximately half of them obtained adequate levels of emotional attention, clarity, and repair. Table 2 identifies the levels of each of the PEI dimensions. With regard to the category rated “To improve”, it is observed that there is a percentage close to 45% in the attention dimension (resulting from the categories “Insufficient” and “Excessive”), 25% in the repair dimension, and almost 40% in the case of emotional clarity. Regarding comparisons between boys and girls, the results showed statistically significant differences with a small effect size in the three subscales studied: Attention (*p* = 0.018, V = 0.156); Clarity (*p* = 0.008, V = 0.170), and Repair (*p* = 0.008, V = 0.171). The percentage of males with excessive attention was higher, as well as with adequate levels in clarity and repair. However, the percentages of excellent clarity and repair were higher among females. No differences were identified regarding sex between those who need to improve in any of the three dimensions.

Table 3 presents the results of the three emotional intelligence dimensions (Attention, Clarity, and Repair) according to the academic year. In this case, the significance tests showed no significant differences regarding the emotional Attention, Clarity, and Repair dimensions among the students of the different courses (see *p* values in Table 2). However, despite no significant differences in the Clarity dimension, it was observed that the effect size (see Tau-c value in Table 2) could be classified as a small effect size.

### 3.2. Emotional Intelligence and Academic Performance

An analysis of the relationship between emotional intelligence and student performance (assessed according to their mean grades) shows no differences in any of the categorised subcomponents of emotional intelligence, as can be seen in Table 4. ANOVA results showed no statistical significance associated to any of the dimensions, thus allowing to reject normality, nor homoscedasticity, *p* > 0.05 in all cases.

### 3.3. Emotional Intelligence and Emotional Well-Being

With regard to the relationship between the emotional intelligence dimensions and emotional well-being, once assumed normality and homoscedasticity of the dimensions (*p* > 0.05 in all cases), statistically significant differences are found regarding the Clarity and Repair dimensions (see Table 5). A post-hoc analysis of the Clarity and Repair dimensions allowed to detect, based on Tukey’s range test, significant differences between the category “To improve” and “Adequate” and “Excellent” (*p* < 0.001 in all cases). No significant differences were found for the clarity dimension between the “Excellent” and “Adequate” categories (*p* = 0.508), but these were found for the repair dimension (*p* < 0.001). When analysing the effect size of these differences, it is observed that, in the Clarity dimension, a moderate effect is found between the categories “To improve” vs. “Adequate” (Cohen’s *d* = 0.56); a small size effect between the categories “Adequate” vs. “Excellent” (Cohen’s *d* = 0.22); and moderate but close to large effect size between the categories “To improve” vs. “Excellent” (Cohen’s *d* = 0.78). As for the Repair dimension, a moderate effect size is found between the categories “To improve” vs. “Adequate”, and between “Adequate” vs. “Excellent” (Cohen’s *d* = 0.61 and 0.62, respectively), and a large effect size between “To improve” vs. “Excellent” (Cohen’s *d* = 1.11).

## 4. Discussion

The objective of this study was to analyse the levels of emotional intelligence and their relationship with academic performance and emotional well-being among students of compulsory secondary education of an associated educational centre.

First, it is noted that the most participating students are placed above the adequate levels in the emotional Attention, Clarity, and Repair dimensions, although it is true that the percentages associated with the category rated “To improve” are high in absolute terms (44.71%, 39.9% and 24.3% for attention, clarity and repair, respectively). The obtained results resemble previous research conducted by Guerra-Bustamante et al. [33] on a sample of 646 Spanish students in the same educational stage. This study obtained percentages of students in the “To improve” category in the three dimensions of the TMMS-24, in particular 41.0%, 39.39%, and 29.4% for attention, clarity, and repair, respectively.

Second, it is concluded that there are statistically significant differences regarding emotional intelligence levels between sample boys and girls for all three dimensions. There are similarities with the findings of Serrano and Andreu [26] and Trigoso [36], who found higher scores for girls in the Attention component, while boys scored higher in Repair. Likewise, a study performed on Chilean young population also identified significantly higher levels among girls for the attention to the feelings dimension [37]. In our study, these findings are not confirmed for the Attention dimension, since despite the fact that approximately half of both groups (boys and girls) are in the “Adequate” category, there is a greater presence of boys in the “Excessive” category and girls in the “Insufficient” category. However, as regards the Repair dimension, there are similarities with the findings of Serrano and Andreu [26] and Trigoso [36], as the percentage of boys reporting Adequate and Excellent scores on this component is higher than that of girls. Regarding the results of these studies, Pérez-Bonet and Velado-Guillén [38] stated that there are two tendencies: on the one hand, greater self-attention is paid to women’s feelings compared to men and, on the other hand, men have an advantage in the emotional repair variable, although they consider these trends unsteady and contradictory today, as it can be partially seen in the present study. Another research conducted on samples of adolescents found no significant differences between boys and girls regarding the Clarity and Repair dimensions [39]. This discrepancy with the results of our study could be explained by the imbalance in the distribution of our sample with respect to gender, with boys being more frequent.

In relation to course comparisons, no differences were found between students of different courses for any of the emotional intelligence dimensions studied, as it also occurs in some other studies [26]. The study by Alhabi on emotional intelligence in Saudi school-age children did not either find significant differences regarding courses [40]. However, these findings contrast with those stated by other authors who found that emotional intelligence is lower in 2nd course of ESO than in the other educational levels and, in particular, significantly lower than in 4th course of ESO [16]. However, it should be noted that the instrument for measuring emotional intelligence used in this study (Emotional Quotient—Youth Version) was different from the one used in our study, so the assessment of the phenomenon could differ.

Considering the study of the relationship between the dimensions of emotional intelligence and academic performance, our findings do not support the existence of a relationship between them. These results differ from those observed by other researchers who did find statistically significant positive correlations between emotional intelligence and academic performance, although with different effects as compared to the results found in this study [13,14,41]. While this is true, these authors used different instruments for the measurement, in this case, MSCEIT [42] for the first two [14,41], and TEIQue-ASF [43] in the third one [13]. Suleman et al. studied the association between emotional intelligence and academic success in young Pakistanis. The results revealed that students with a higher level of emotional intelligence achieved better academic results. In addition, academic success was strongly associated with the managing relations, integrity, and self-development dimensions, and moderately associated with self-awareness, empathy, self-motivation, emotional stability, value orientation, commitment, and altruistic behaviour [44]. McCann et al.’s meta-analysis concluded that emotional intelligence is a predictive factor for academic success, especially in humanities-related areas of knowledge. These authors identified three elements that underpin the link between emotional intelligence and academic success: (a) regulating academic emotions; (b) building social relationships at school; and (c) academic content overlap with emotional intelligence [45]. Our results may differ by the fact that academic performance considered as a categorical variable rather than continuous. In any case, the authors of the meta-analysis recommend conducting longitudinal studies that consolidate the role of these three mechanisms in the relationship between IE and academic performance.

When focusing on the work used by the TMMS-24, Jiménez-Morales and López-Zafra [46], in a study on ESO students, they found a correlation between the attention and repair subscale and academic performance, though not for the Clarity dimension. Buenrostro-Guerrero et al. [47], in a study on a sample of first-year high school students, observed a positive and significant correlation with academic performance only for the TMMS-24 Repair dimension. In this same research, another emotional intelligence assessment tool was used, based on the Bar-On theoretical model [48], finding correlations between academic performance and most of the subscales. Instead, results have been found matching those from our study regarding students from last course, where no significant association was observed between emotional intelligence, evaluated through TMMS-24, and the academic variables mean grade and number of fails [4,26]. This dichotomy can be explained by the difficulty in conceptually defining emotional intelligence [49]. Baudry et al review of trait emotional intelligence, as measured with TMMS, revealed a greater association with better mental health than with physical and overall health. In addition, intrapersonal dimensions, and especially the regulation of emotions, have stronger health effects than interpersonal dimensions [50].

Finally, after examining the relationship between emotional intelligence and emotional well-being of high school students, the results indicated statistically significant differences in emotional well-being based on students’ outcomes in Clarity and Repair, finding bigger effect sizes (Cohen’s d) among the categories rated “To improve” and “Excellent”. These results were consistent with most of the revised literature. Other authors also obtained significant correlations between personal well-being and the Clarity and Repair dimensions [51], but not in the emotional attention component, on a sample of students aged 11–12 [4]. Serrano and Andreu [26] found that the emotional attention dimension of adolescents was negatively associated with subjective well-being, and positively related with perceived stress, while with clarity and emotional repair the opposite happened, as they positively correlated with subjective well-being, and negatively with perceived stress. Gascó et al. [52] found that the emotional Clarity and Repair subscales were positively related to vital satisfaction of adolescents, and negatively associated with perceived stress and somatic complaints, though emotional attention showed the opposite pattern. More recently, Guerra-Bustamante et al. [33], on a sample of students from the four high school courses, found that the Clarity and Repair components positively correlated with happiness/personal well-being, finding no relationship with the attention component, which are very similar results to those of the present research.

Some limitations should be considered in the interpretation of the results of this study. First, the cross-sectional design used does not allow identifying causal relationships between the studied variables, so it would be interesting to carry out future longitudinal studies. Nevertheless, other studies like Palomera et al. [53] show that the ability to perceive emotions is a stable predictor of less clinical and emotional mismatch and greater personal adjustment. Another limitation may be the incidental nature of the sample used, limited to particular socio-economic characteristics and whose size limits the generalisation of the results. The limited approach to the phenomenon should also be mentioned, as it does not include the perspective of parents and teachers of emotional intelligence. If the peer measure of emotional intelligence is also considered it would give a larger picture of the phenomena.

## 5. Conclusions

A significant percentage of the students have been detected with aspects to improve in the field of emotional intelligence. Specifically, 45% of students were found to be at inadequate levels in the Attention dimension (25% for “Insufficient” and almost 20% “Excessive”), 40% in the Clarity dimension, and 25% in the Repair dimension.

Similarly, there seems to be a clear and positive relationship between the Clarity and Repair dimensions and well-being, whereas the role of Attention does not tend to be significant or even has negative effects. In this sense, the results suggest a greater need for support from girls in the Attention below the level) and Repair dimension; and boys in the attention (over the level) and Clarity dimensions, for all courses.

This study did not support the previous findings which revealed a relationship between emotional intelligence and academic performance. The complexity of the phenomenon itself, its multiple definitions and interpretations favor the disparity in the results of the studies. However, a relationship between well-being and emotional intelligence is seen. Specifically, it is confirmed that understanding one’s emotions (emotional clarity) allows one to face vital events more openly and positively, increasing the emotional well-being. Similarly, adequately regulating emotions (emotional repair) minimises unpleasant emotions, allowing the person to experience pleasant emotional states most of the time.

## Figures and Tables

**Table 1:** Interpretation of the Trait Meta-Mood Scale (TMMS)-24 scores.

Dimension	Male	Female
Attention	To be improved: Little attention <21	To be improved: Little attention <24
	Adequate attention from 22 to 32	Adequate attention from 25 to 35
	To be improved: Too much attention >33	To be improved: Too much attention >36
Clarity	To be improved <25	To be improved <23
	Adequate from 26 to 35	Adequate from 24 to 34
	Excellent >36	Excellent >35
Repair	To be improved <23	To be improved <23
	Adequate from 24 to 35	Adequate from 24 to 34
	Excellent >36	Excellent >35

**Table 2:** Analysis results of the emotional Attention, Clarity, and Repair dimensions for the whole sample and according to sex.

			Sex						
	Total *n* = 333		Male *n* = 191		Female *n* = 142				
	f	% (CI)	f	% (CI)	f	% (CI)	χ^2^	*p*	Cramer’s V
**Attention**							8.082	0.018	0.156
Insufficient	83	24.92% (20.27–29.57)	40	20.9% (15.2–26.68)	43	30.3% (22.71–37.89)			
Adequate	184	55.25% (49.90–60.60)	104	54.5% (47.42–61.58)	80	56.3% (48.11–64.49)			
Excessive *	66	19.81% (15.52–24.10)	47	24.6% (18.48–30.72)	19	13.4% (7.78–19.02)			
**Clarity**							9.591	0.008	0.170
To improve	133	39.9% (34.64–45.16)	72	37.7% (30.81–44.59)	61	43.0% (34.83–51.17)			
Adequate *	169	50.75% (45.37–56.13)	108	56.5% (49.45–63.55)	61	43.0% (34.83–51.17)			
Excellent *	31	9.39% (6.25–12.53)	11	5.8% (2.48–9.12)	20	14.1% (8.36–19.84)			
**Repair**							9.685	0.008	0.171
To improve	81	24.3% (19.69–28.91)	39	20.4% (14.67–26.13)	42	29.6% (22.07–37.13)			
Adequate *	216	64.9% (59.77–70.03)	137	71.7% (65.29–78.11)	79	55.6% (47.40–63.80)			
Excellent *	36	10.8% (7.46–14.14)	15	7.9% (4.06–11.74)	21	14.8% (8.94–20.66)			

* Percentages differences by sex.

**Table 3:** Analysis results for the emotional Attention, Clarity, Repair dimensions according to the course of the students.

	Course										
	1st ESO * n = 86		2nd ESO * n = 80		3rd ESO * n = 86		4th ESO * n = 81				
	f	% (IC)	f	% (IC)	f	% (IC)	f	% (IC)	χ^2^	*p*	Tau-c
**Attention**									4.633	0.592	0.015
Insufficient	24	27.9%	17	21.3%	24	27.9%	18	22.2%			
		(18.37–37.43)		(12.27–30.33)		(18.37–37.43)		(13.09–31.31)			
Adequate	45	52.3%	49	61.3%	41	47.7%	49	60.5%			
		(41.68–62.92)		(50.56–72.04)		(37.08–58.32)		(49.79–71.21)			
Excessive	17	19.8%	14	17.5%	21	24.4%	14	17.3%			
		(11.33–28.27)		(9.12–25.88)		(15.27–33.53)		(9.01–25.59)			
**Clarity**									7.723	0.259	−0.109
To improve	30	34.9%	25	31.3%	40	46.5%	38	46.9%			
		(24.77–45.03)		(21.07–41.53)		(35.90–57.10)		(35.96–57.84)			
Adequate	45	52.3%	47	58.8%	40	46.5%	37	45.7%			
		(41.68–62.92)		(47.95–69.65)		(35.90–57.10)		(34.78–56.62)			
Excellent	11	12.8%	8	10.0%	6	7.0%	6	7.4%			
		(5.70–19.90)		(3.38–16.62)		(1.58–12.42)		(1.66–13.14)			
**Repair**									9.646	0.140	−0.071
To improve	23	26.7%	15	18.8%	21	24.4%	22	27.2%			
		(17.30–36.10)		(10.18–27.42)		(15.27–33.53)		(17.45–36.95)			
Adequate	49	57.0%	54	67.5%	57	66.3%	56	69.1%			
		(46.48–67.52)		(57.17–77.83)		(56.25–76.35)		(58.97–79.23)			
Excellent	14	16.3%	11	13.8%	8	3.7%	3	3.7%			
		(8.45–24.15)		(6.19–21.41)		(0–7.71)		(0–7.84)			

* ESO: Compulsory Secondary Education.

**Table 4:** Comparison of means in academic performance based on the emotional Attention, Clarity, and Repair dimensions.

	*n*	M	SD	95% CI	F	*p*
**Attention**					0.524	0.593
Insufficient	83	3.34	1.10	3.10–3.58		
Adequate	184	3.20	1.06	3.05–3.36		
Excessive	66	3.29	1.08	3.03–3.56		
**Clarity**					0.026	0.975
To improve	133	3.24	1.12	3.05–3.44		
Adequate	169	3.27	1.05	3.11–3.43		
Excellent	31	3.24	1.02	2.86–3.61		
**Repair**					0.956	0.385
To improve	81	3.16	1.10	2.92–3.41		
Adequate	216	3.26	1.07	3.11–3.40		
Excellent	36	3.46	1.05	3.14–3.37		

TMMS: Scale: 1 (do not agree)–5 (totally agree). Academic performance: Scale: 1 (fail)–5 (excellent).

**Table 5:** Comparison of emotional well-being means based on the emotional Attention, Clarity, and Repair dimensions.

	*n*	M	SD	95% CI	F	*p*
**Attention**					0.020	0.980
Insufficient	83	36.78	5.52	35.57–37.98		
Adequate	184	36.63	6.06	35.74–37.51		
Excessive	66	36.68	5.45	35.34–38.02		
**Clarity**					14.922	<0.001
To improve	133	34.67	5.78	33.68–35.66		
Adequate	169	37.82	5.55	36.97–38.66		
Excellent	31	39.03	4.65	37.32–40.74		
**Repair**					21.068	<0.001
To improve	81	33.74	6.14	32.38–35.09		
Adequate	216	37.14	5.34	36.43–37.86		
Excellent	36	40.47	5.80	38.91–42.02		

TMMS: Scale: 1 (do not agree)–5 (totally agree). KIDSCREEN-10: Scale: 1 (not at all/never)–5 (extremely/always), total scores range from 10 to 50, and higher scores indicate higher levels of emotional well-being.
